# Impact of Forrest H. Nielsen on my Career

**DOI:** 10.1007/s12011-026-04990-1

**Published:** 2026-01-24

**Authors:** Jerry W. Spears

**Affiliations:** Department of Animal Science, North Carolina State University, Raleigh, NC 27695 USA

**Keywords:** Nickel, Boron

## Abstract

Dr. Forrest (Frosty) H. Nielsen has been a pioneer in the area of ultratrace elements for over 50 years. His research contributions, especially with nickel and boron, have stimulated much interest and additional research on these two trace minerals. This paper will discuss how Dr Nielsen’s research has impacted my career.

Dr. Forrest (Frosty) H. Nielsen has been a pioneer in the area of ultratrace element research in mammals. According to Research Gate he has published 328 articles and his research has been cited over 22,000 times. His early research with nickel (Ni) opened doors for further research demonstrating functional roles for Ni in plants and bacteria.

Dr. Nielsen’s research greatly impacted my career and influenced several of my graduate students. In the spring of 1976, I was searching for a dissertation research topic. My advisor, Dr E. E. Hatfield, had considerable funding which allowed me to pursue areas of research many students would not have the opportunity to explore. In my search for a research topic, I came across two papers [1, 2] authored by Dr Nielsen in the Journal of Nutrition that caught my interest. The papers were “Nickel deficiency and nickel – rhodium interaction in chicks” and “Nickel deficiency in rats”. The Journal of Nutrition papers cited two other papers [3, 4] that I read extensively. These papers resulted in me choosing a Ph.D. research topic of “Studies on the Role of Nickel in Animal Nutrition”. Little did I know at the time that I would continue research in this area for 13 years, as a graduate student and later in my professional career.

My initial studies [5, 6] with lambs fed a semi-purified diet low in Ni, focused largely on variables that Dr. Nielsen had found to be affected by Ni deficiency in rats and chicks, such as oxygen uptake by liver, liver cholesterol and lipid concentrations, hematocrit, and uptake of ^63^Ni by tissues following oral or intraperitoneal administration. In 1975 Jack-bean urease was shown to be the first Ni metalloenzyme. This led us to evaluate rumen bacterial urease in lambs fed low Ni (60 ng/g) or Ni supplemented (5 µg/g) diets. We found that ruminal urease actively was undetectable or extremely low in lambs fed low Ni diets and was increased by 8 to 9-fold by Ni supplementation [7]. These finding indicated for the first time that bacterial urease was a Ni-dependent enzyme. It had been suggested that bacterial urease present in the rumen epithelial tissue greatly facilitated the transfer of urea-nitrogen from the blood across the rumen wall [8]. Nitrogen recycling to the rumen is of major importance in ruminants consuming low protein diets. Based on this hypothesis, we decided to examine the effect of dietary Ni on rumen epithelial urease activity and performance of lambs and steers fed diets low or adequate in protein [9, 10]. Our research at that time switched from using semi-purified diets to more practical corn-based diets, considerably higher in Ni (0.31–0.85 µg/g). Rumen epithelial urease activity was increased by Ni supplementation in lambs [9], and performance of lambs and steers tended to be improved by Ni supplementation of diets low in protein, but not in diets adequate in protein.

I resumed research with Ni as an Assistant Professor at North Carolina State University in 1981. My first graduate student (Scott Starnes) conducted his research on the interaction between Ni and protein source and level on rumen urease activity and performance of steers [11]. His research indicated that protein source influenced responses to dietary Ni. In the early 1980’s Ni was shown to be an essential component of coenzyme factor F_430_, which is involved in the terminal reaction of methanogenesis by methanogenic bacteria. Tom Oscar’s Ph.D. research following up on this finding by examining the effect of dietary Ni on in vitro and in vivo methanogenesis by ruminal microorganisms [12, 13]. Oscar demonstrated that ^63^Ni was incorporated into factor F_430_ in ruminal microorganisms [14].

Later, Todd Armstrong followed up on Dr. Nielsen’s earlier boron (B) research in chicks and humans [15, 16] by conducting his Ph.D. research on B in swine nutrition. Dr. Nielsen collaborated on many of Todd’s studies. Todd’s research indicated that B supplementation altered certain bone characteristics, improved gain, and decreased the inflammatory response in pigs following intradermal injection of phytohemagglutinin in pigs fed corn-based diets supplemented with animal protein sources, know to be lower in B than plant protein sources [17–19]. In a long-term study female pigs were supplemented with 0, or 5 µg B/g diet from weaning through sexual maturity, breeding, gestation, and lactation [20]. Boron supplementation did not affect pregnancy rate or number of pigs born alive, but increased pig weaning weight at 21 d of age. In a limited number of gilts sacrificed at 35 d of gestation, B supplementation greatly increased B concentrations in uterus, oviduct, ovary, embryo, and muscle [20]. Scott Fry following up on our swine studies, with his M.S. research evaluating the effect of dietary B on immune function and responses to a viral respiratory disease challenge in steers [21, 22]. Boron addition had minimal effects on immune responses and clinical signs of respiratory disease following disease challenge, likely due to the much higher concentrations of B (7.2 to 13.2 µg/g DM) in the practical ruminant diets fed.

All of my graduate students who conducted research with Ni or B based on Dr Nielsen’s earlier research findings have excelled in their careers. Scott Starnes is Vice President and General Manager of Eastern Minerals, Inc. Tom Oscar is a Research Food Technologists with the USDA Agricultural Research Service. Todd Armstrong is currently President North America Region at Phibro Animal Health, and Scott Fry is Director of the Swine Business Unit at Phibro Animal Health.

I first met Dr. Nielsen at the Federation Meetings (now Experimental Biology Meetings) in 1978. He asked me a question after my presentation and later visited with me and provided encouragement. A graduate student can always use encouragement and it was great to meet a man I greatly admired and respected. Frosty and I interacted on many occasions over the next 30 years and he always was such an encourager to me. We both attended and participated at trace mineral symposiums on Ni and other trace elements in the former East Germany in 1980 and 1986. Later we presented invited presentations at a B symposium in China. We both served on the National Council Research committee on “Minerals and Toxic Substances in Diets and Water of Animals”. Members of this committee wrote the Mineral Tolerance of Animals book that was published in 2005. Frosty Nielsen has been a mentor, research collaborator, and friend to me for many years. I wish him the best in his well-deserved retirement.

## Data Availability

No datasets were generated or analysed during the current study.
